# Changes in lipid composition associated with electronic cigarette use

**DOI:** 10.1186/s12967-020-02557-9

**Published:** 2020-10-07

**Authors:** Holly R. Middlekauff, Kevin J. William, Baolong Su, Kacey Haptonstall, Jesus A. Araujo, Xiaohui Wu, Jason Kim, Tamer Sallam

**Affiliations:** 1 Division of Cardiology, Department of Medicine, University of California, Los Angeles, CA USA; 2 Molecular, Cellular and Integrative Physiology Program, University of California, Los Angeles, CA USA; 3 Department of Biological Chemistry, University of California, Los Angeles, CA USA; 4 Molecular Biology Institute, University of California, Los Angeles, CA USA

**Keywords:** Lipids, Tobacco, Electronic cigarettes

## Abstract

**Background:**

Electronic cigarette use is on the rise despite a number of reports linking electronic cigarettes with adverse health outcomes. Recent studies have suggested that alterations in lipid signaling may be one mechanism by which electronic cigarettes contribute to lung pulmonary function. Vitamin E acetate, for example, is synthetic form of Vitamin E transported via lipids, found to be associated with electronic cigarette associated lung injury. Lipids are absolutely critical for normal lung physiology and perturbations in a number of lipid pathways have been associated with respiratory illness. Is it conceivable that electronic cigarette use even in seemingly healthy cohorts are associated with alterations in lipid pathways?

**Methods:**

To investigate quantitative alterations in the plasma lipidome associated with electronic cigarette use in healthy we obtained plasma samples from 119 male and female participants with who were either: (1) chronic tobacco cigarette (TC) smokers (> 12 months of self-reported TC use), (2) chronic Electronic cigarette (EC) users (> 12 months of self-reported EC use), or (3) non-users. We measured quantitative lipid species across different lipid sub-classes from plasma samples using the Sciex Lipidyzer.

**Results:**

We found that male and female tobacco and electronic cigarette users had distinct lipidome signatures across a number of lipid species although the vast majority of lipids were unchanged when compared to non-users. Intriguingly, we found that female but not male electronic cigarette users had lower levels of plasmalogens, critical glycerophospholipids secreted by alveoli and required for normal surfactant function.

**Conclusions:**

In summary, our study does not reveal striking changes associated with electronic cigarette use but we observed sex-specific changes in lipids known to be critical for lung function.

## Background

Electronic cigarettes (ECIGS) were originally introduced in 2004 as a safe alternative to tobacco cigarettes (TCIGS). In less than a decade from their introduction, ECIG use has increased by 5000% and recent estimates project that ECIG use will surpass TCIG use in two decades [1–3]. More recently, a rapidly growing number of reports have linked ECIG use with a number of adverse health events including acute or subacute lung disease [4–6]. The pattern of injury appears to be distinct from adverse effects of TCIGs on lung disease lending weight to the notion that liquid constituents of ECIGS such as propylene glycol, glycerin, flavorant(s) or other components beyond nicotine may be involved in mechanisms of lung injury.

Significant heterogeneity is observed in the pattern and severity of lung injury associated with ECIG use which hints that diverse mechanisms may be at play. Although the precise mechanism(s) of ECIG induced lung injury remain poorly understood, there are two prevailing theories. First, that components of ECIGS cause a direct chemical injury to the lungs [7]. A second hypothesis is that ECIG use is associated with alterations in normal lung physiology that predispose to lung disease [8]. In a recent preclinical study in female mice, ENDS exposure led to alterations in lung phospholipid composition and innate immune responses independent of nicotine [9]. Intriguingly, a subset of vaping associated pulmonary diseases have been associated with lipid overload of immune cells and more recent evidence suggested that changes in specific lipids such as the isoprenoid Vitamin E acetate is associated with acute lung injury [8, 10]. The later phenomenon is often referred to as E-cigarette or Vaping Product Use-Associated Lung Injury (EVALI) and is thought to be uncommon in vapers who do not vape THC. Interestingly, liquid components of ECIGS such as propylene glycol and glycerol can theoretically interfere with critical lipid metabolic reactions but whether ECIG use in humans is associated with perturbations in lipid homeostasis and how ECIG use compare with TCIG use remains unexplored.

Lipids are critical for normal lung physiology since they play a direct role in membrane structure integrity, cell-signaling, oxidative stress responses and surfactant synthesis [11, 12]. For example, perturbations in a glycerophospholipid known as plasmalogen secreted by pneumocytes has been associated with pulmonary diseases [13]. In this study of otherwise healthy, young males and females, we aimed to investigate quantitative alterations in plasma lipidome associated with chronic ECIG use compared to chronic TCIG use and non-smoking. We validate some of the previously observed effects of TCIG use on lipid composition and find that ECIG use is associated with unique sexually dimorphic signatures in lipid species that is independent from nicotine use. Notably, we observe that ECIG use in females is associated with lower level of lipids critical for surfactant function.

## Methods

Plasma samples were obtained during the period of 2015–2018 in a cohort of subjects participating in our clinical investigations (NCT03072628, NCT02740595, NCT02724241) of tobacco product use.

### Human Subjects

In these studies [14], plasma samples were obtained from healthy male and female subjects, ages 21–45 years, who were: 1) chronic (≥ 12 months) TCIG smokers, 2) chronic (≥ 12 months) ECIG users (no dual users), or 3) non-smokers. End-tidal CO was measured in ECIG users to detect those who were surreptitiously using TCIGs, and if CO > 10 ppm was detected, the subjectwas eliminated from the study. *Exclusion Criteria:* Non-sinus rhythm (e.g. atrial fibrillation); competitive or trained athletes; pregnant women; taking prescription medications regularly (oral contraceptives were allowed); more than 2 alcoholic drinks per day; using illicit drugs, including marijuana, or a positive urine toxicology test; chronic illness, including asthma, hypertension, heart disease, diabetes; hyperlipidemia; obesity (BMI > 30); or regular exposure to secondhand smoke in non-TCIG smokers. The experimental protocol was approved by the Institutional Review Board at the University of California, Los Angeles, and written informed consent was obtained from each participant.

### Experimental protocol

Participants were asked to abstain from food, caffeine, exercise and tobacco product use for at least 12 h prior to the study. Participants were placed in a supine position in a quiet, temperature-controlled (21 °C) room in the Human Physiology Laboratory located in the University of California, Los Angeles Clinical Translational Research Center. Blood was drawn in pre-iced heparinized vacutainers and placed on ice. Blood was centrifuged to separate into plasma samples, which were frozen at − 80 °C.

### Lipid analysis

25 µl of plasma was pipetted into a glass tube for extraction. A modified Bligh and Dyer extraction (Bligh, 1959) is carried out on samples. Prior to biphasic extraction, a 13-lipid subclass Lipidyzer Internal Standard Mix is added to each sample (AB Sciex, 5040156). Following two successive extractions, pooled organic layers are dried down in a Genevac EZ-2 Elite. Lipid samples are resuspended in 1:1 methanol/dichloromethane with 10 mM Ammonium Acetate and transferred to robovials (Thermo 10800107) for analysis. Samples are analyzed on the Sciex Lipidyzer Platform for targeted quantitative measurement of 1100 lipid species across 13 subclasses. Differential Mobility Device on Lipidyzer was tuned with SelexION tuning kit (Sciex 5040141). Instrument settings, tuning settings, and MRM list available upon request. Data analysis performed on Lipidyzer software. Quantitative values were normalized to volume. Samples were analyzed on the Sciex Lipidyzer Platform for targeted quantitative measurement of lipids.

### Statistics

We used ANOVA with correction for statistical analysis of lipid composition between the three groups. Principal component analysis was done using ClusterVis [15]. The input data is the lipid concentration from our lipidomic run measurements and the PCA calculation is based on pcaMethods [15].

## Results

**Fig. 1:**
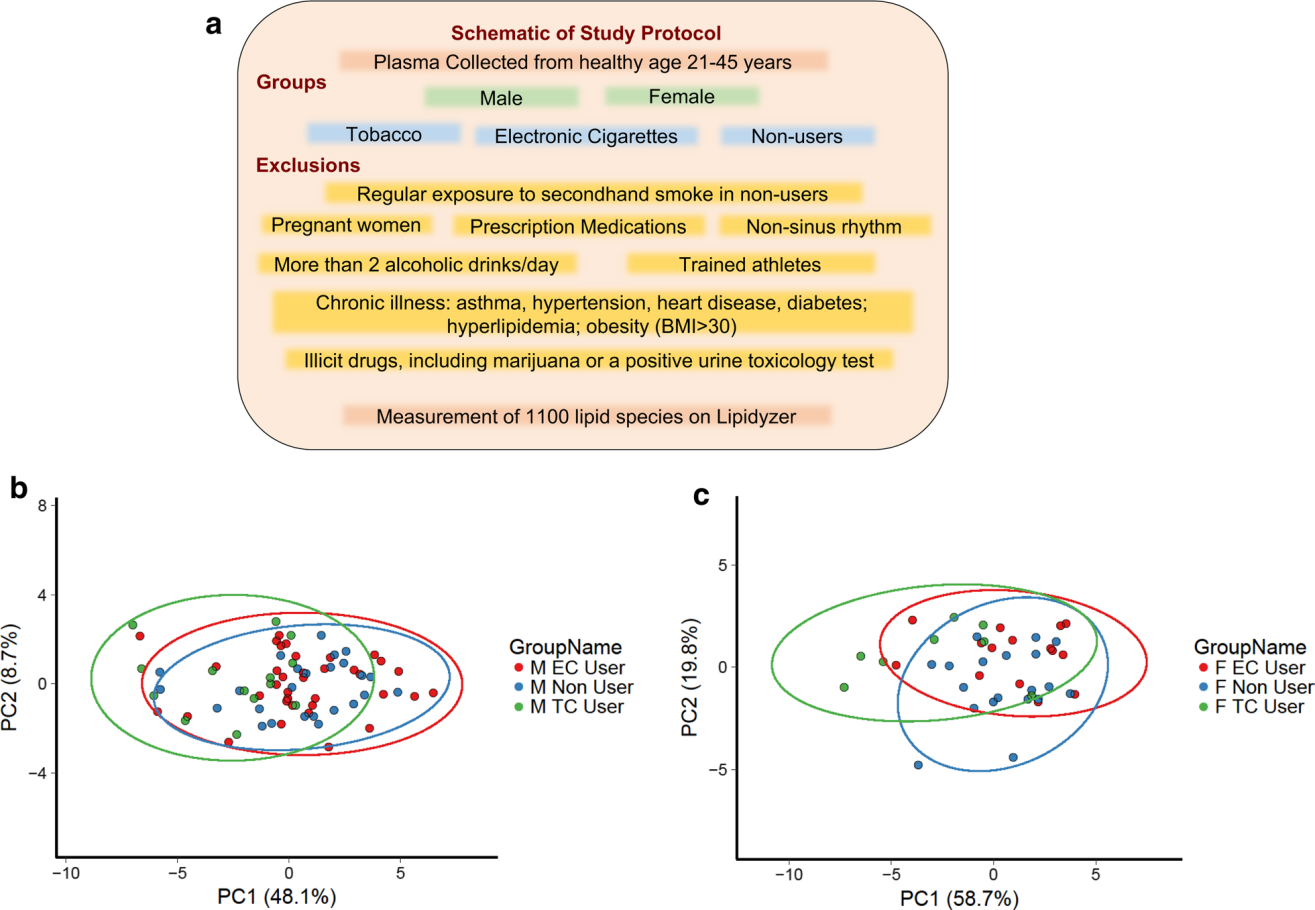
Segregation of lipid profiles by principal component analysis. **a** Schematic of Enrollment strategy. **b** Principal component analysis in male participants showing contribution to variance in lipid measurements across groups. Each dot represents one subject. **c** Principal component analysis in female participants showing contribution to variance in lipid measurements across groups. Each dot represents one subject

Our study enrolled 77 male and 42 female participants. Baseline demographic information of participants is included in Table 1. Targeted serum lipidomics measurement across 1000 different lipid species was performed (Fig. 1a). Principal component analysis (PCA), a technique that summarizes the variation in samples and strong patterns in datasets, showed significant overlap between samples across groups for both males (Fig. 1b) and females (Fig. 1c) consistent with the notion that TCIG or ECIG use do not globally alter lipid composition. For both male and female subjects TCIG users contributed to more variance than ECIG or non-smokers with largest difference in variance across the first component (explains 48% of all variation) between TCIG and non-smokers (Fig. 1).

**Table 1:** Demographic data on study participants

	Control	EC-user	TC-user
N			
Male	26	27	24
Female	19	15	8
Age mean (STD)			
Male	26.4 (4.3)	27.1 (5.1)	27.1 (6.4)
Female	28.9 (7.0)	30 (5.5)	27.5 (4.33)
BMI mean (STD)			
Male	23.6 (2.7)	24.4 (3.7)	25.0 (3.1)
Female	22.9 (2.4)	24.2 (3.4)	23.3 (2.3)
Race if known (N)			
Male			
White	20	12	9
Asian	2	10	10
African American	0	1	2
Hispanic	4	3	3
Native Hawaiian	0	0	0
Female			
White	8	9	5
Asian	5	0	1
African American	1	1	1
Hispanic	0	0	1
Native Hawaiian	2	0	0

**Fig. 2:**
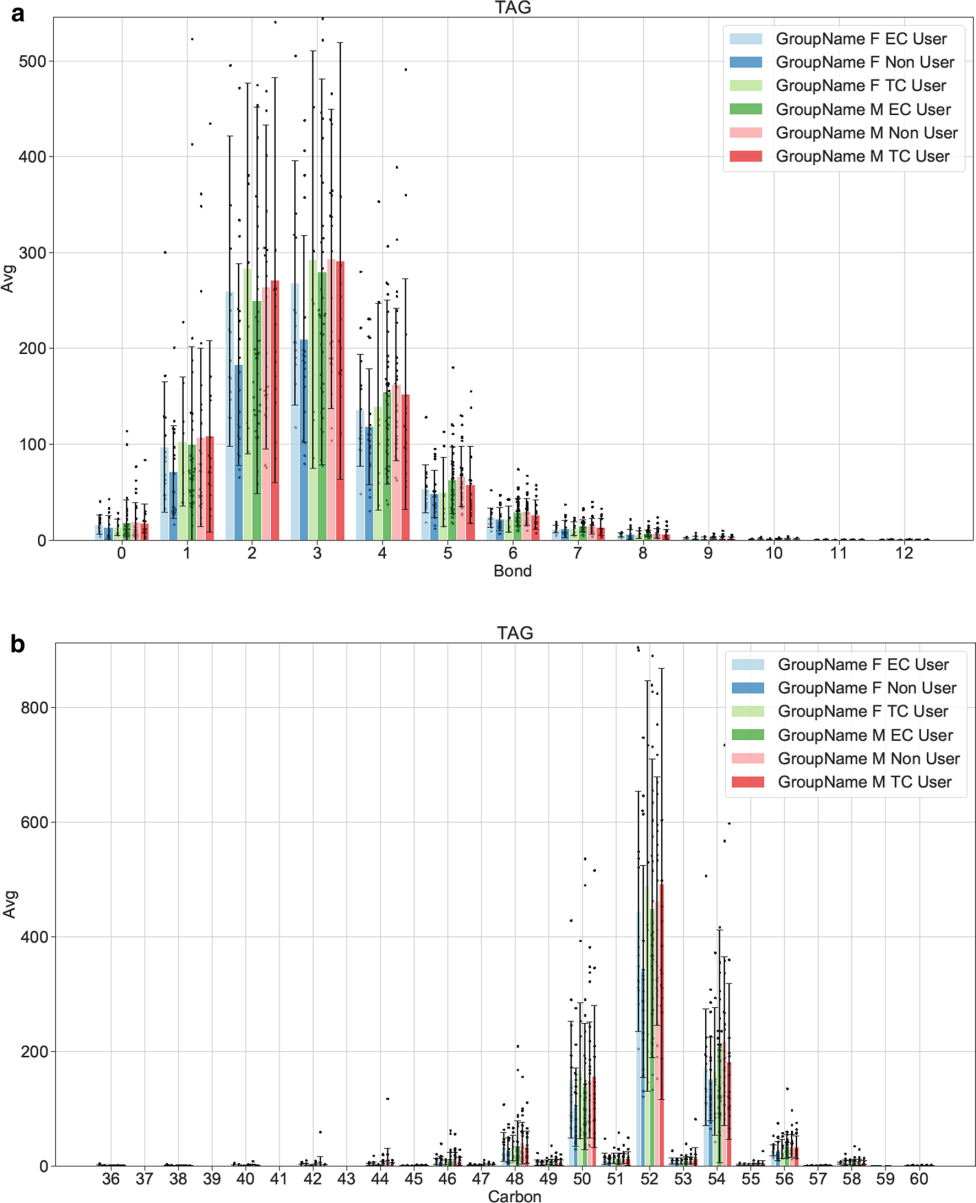
Levels of Triglyceride across groups. Number of **a** Bonds and **b** Carbons for TAG. Each dot is individual samples measured from plasma. Bar graph is mean ± SEM

**Fig. 3:**
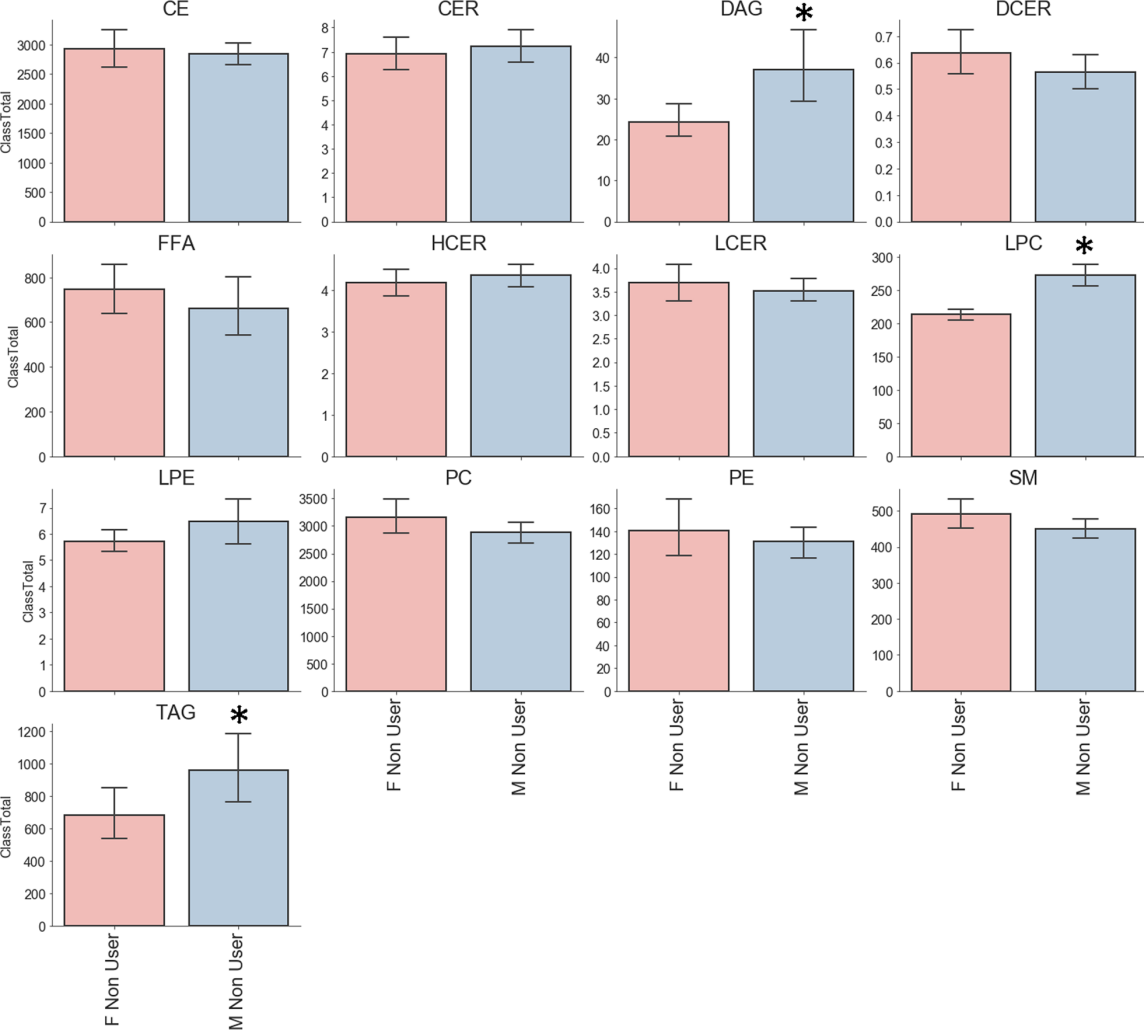
Major lipid classes from comparing male and female non-users. Each bar graph represents mean ± STD. *P < 0.01. *CE* cholesterol ester, *CER* Ceramides, *DAG* diacylglyceroles, *DCER* dihydroceramides, *FFA* free fatty acids, *HCER* hexosylceramides, *LCER* lactosylceramides, *LPC* lysophosphatidylcholine, *LPE* lysophosphatidylethanolamine, *PC* phosphatidylcholine, *PE* phosphatidylethanolamine, *SM* Sphingomyelin, and *TAG* triacylglycerols

**Fig. 4:**
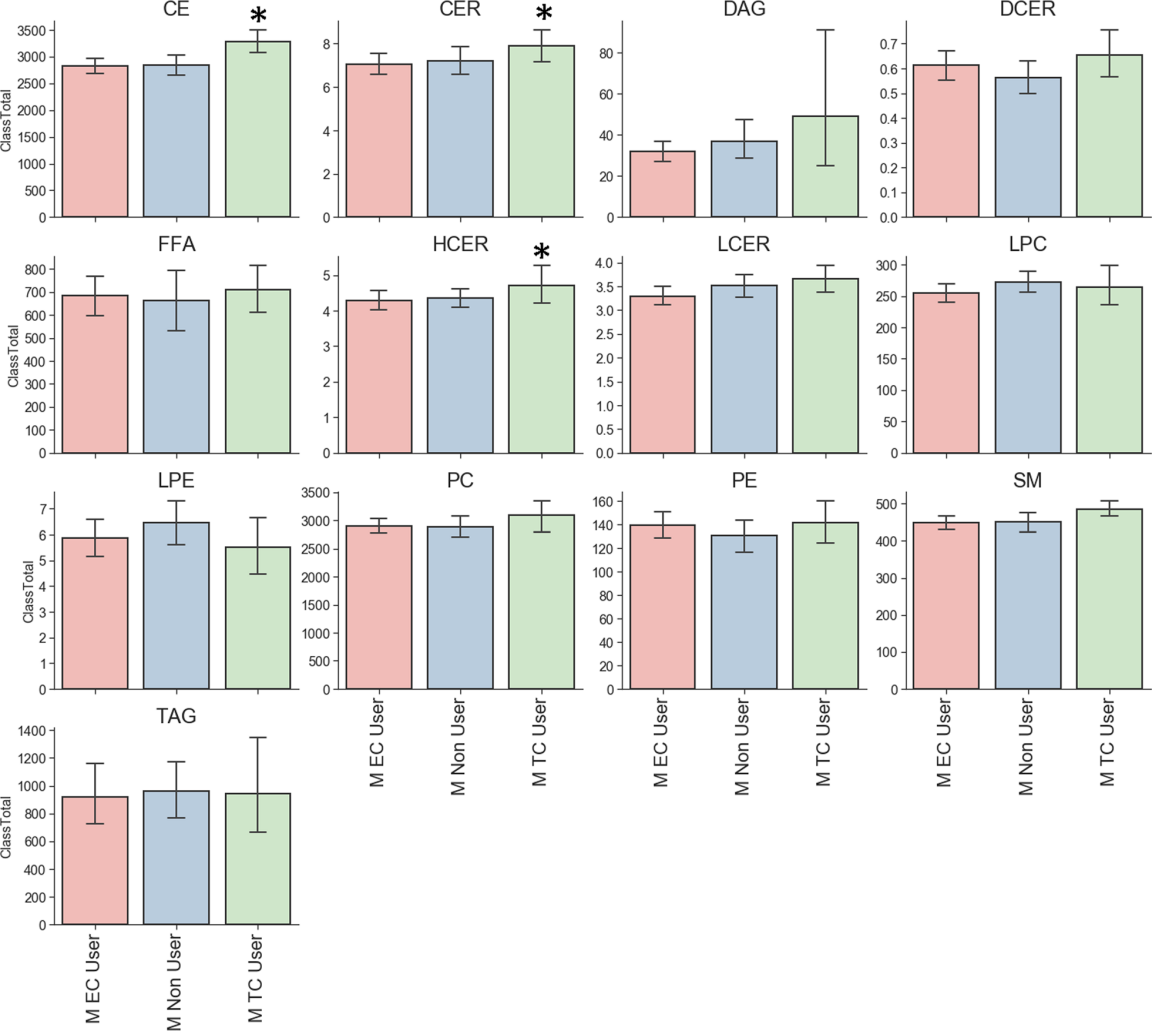
Major lipid classes from serum samples in male participants. Each bar graph represents mean ± STD. *P < 0.01. *CE* cholesterol ester, *CER* ceramides, *DAG* diacylglyceroles, *DCER* dihydroceramides, *FFA* free fatty acids, *HCER* hexosylceramides, *LCER* lactosylceramides, *LPC* lysophosphatidylcholine, *LPE* lysophosphatidylethanolamine, *PC* phosphatidylcholine, *PE* phosphatidylethanolamine, *SM* sphingomyelin, *TAG* triacylglycerols

**Fig. 5:**
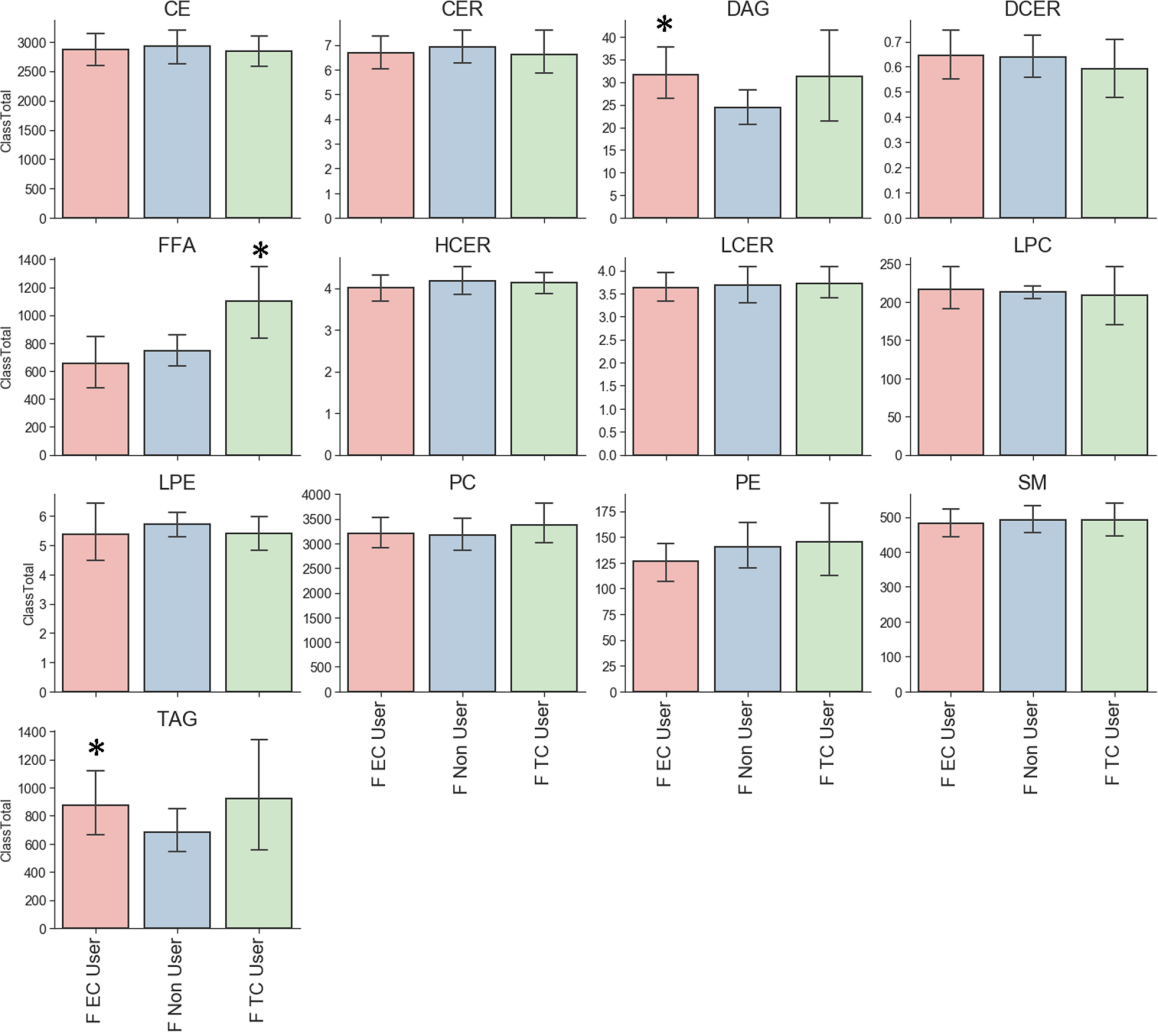
Major lipid classes from serum samples in female participants. Each bar graph represents mean ± STD. *P < 0.01. *CE* cholesterol ester, *CER* ceramides, *DAG* diacylglyceroles, *DCER* dihydroceramides, *FFA* free fatty acids, *HCER* hexosylceramides, *LCER* lactosylceramides, *LPC* lysophosphatidylcholine, *LPE* lysophosphatidylethanolamine, *PC* phosphatidylcholine, *PE* phosphatidylethanolamine, *SM* sphingomyelin, and *TAG* triacylglycerols

Sample distribution of bonds and carbon group for lipid is shown in Figs. 2, 3. Analysis of major classes of lipids across species showed sex-specific trends. In line with previous studies, female and male non-users showed differences in levels of diacylglycerol (DAG), triacylglycerol (TAG) and Lysophosphatidylcholine (LPC). Consistent with previous studies male TCIG smokers showed an increase in total cholesterol esters (CE) species compared with nonuser and ECIG user groups (Fig. 4) [16]. Male participants also showed an increase in ceramides (CER) and hexosylceramides (HCER) in TCIG groups compared with others (Fig. 4). These results are consistent with previous reports that tobacco increases ceramide biosynthesis due to activation of inflammatory signaling [17, 18]. Female participants showed an increase in diacylglyceroles (DAG) and triacylglycerols (TAG) in the ECIG group (Fig. 5). Similar trends were seen for the TCIG group for DAG and TAG although it did not reach statistical significance. Taken together, our results suggest that the vast majority of lipid species are unchanged comparing TCIG or ECIG users with non-smokers however there are striking sexually dimorphic differences driven by TCIG or ECIG group.

**Fig. 6:**
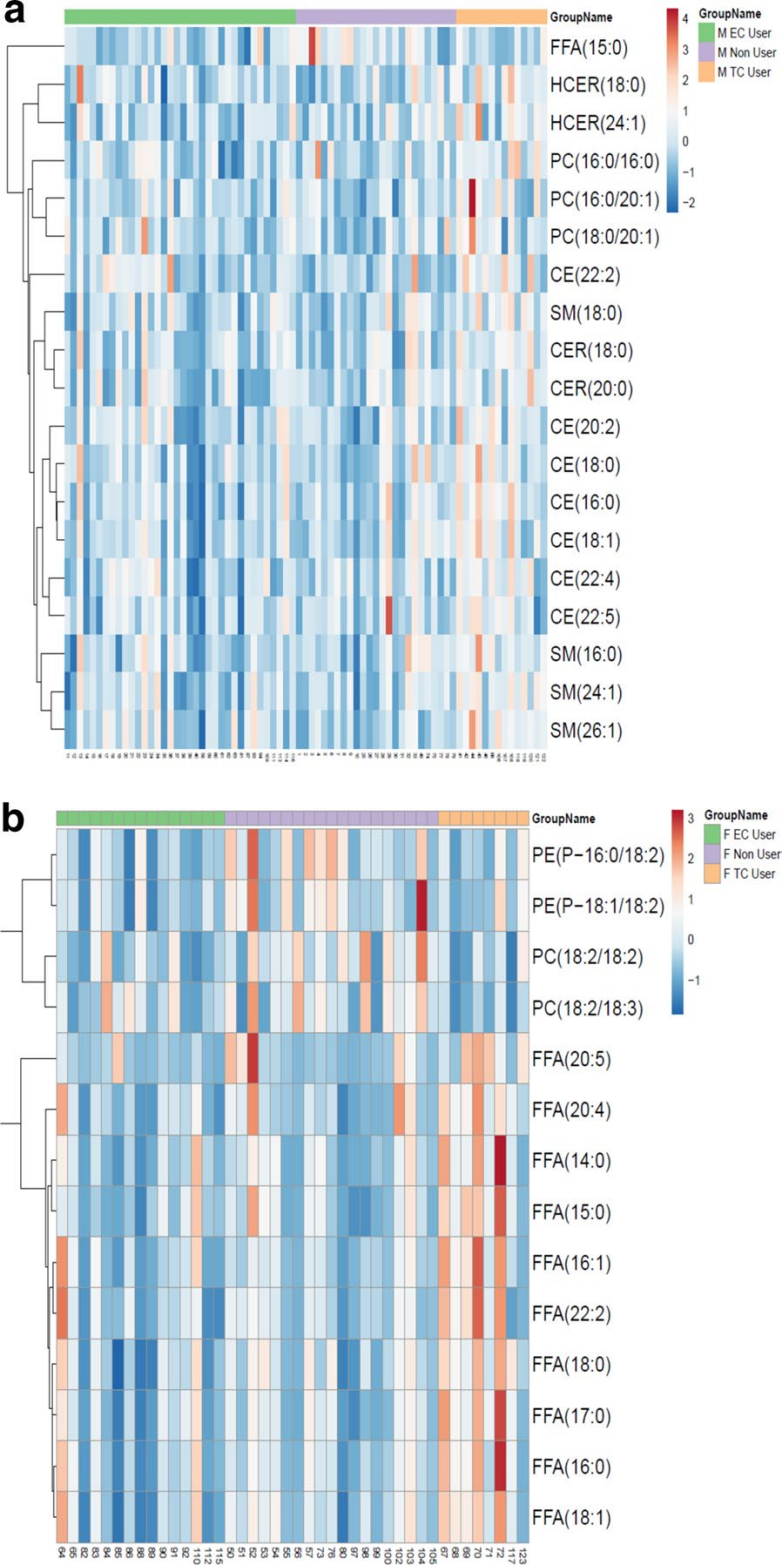
Individual lipid species differentially measured across groups. Heat map for individual lipids measured across groups for A. Male and B. Female participants. Each species here is statistically significant at P < 0.05. *CE* cholesterol ester, *CER* ceramides, *FFA* free fatty acids, *HCER* hexosylceramides, *PC* phosphatidylcholine, *PE* phosphatidylethanolamine, and *SM* sphingomyelin

**Fig. 7:**
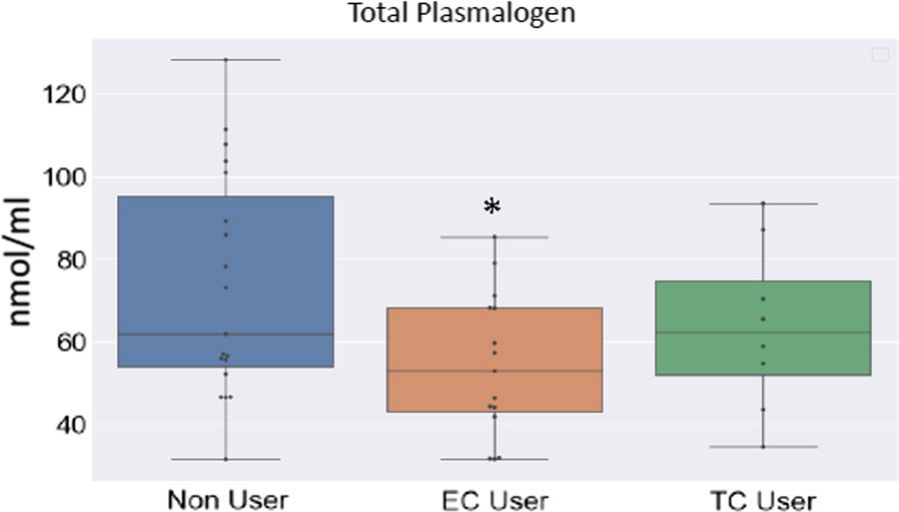
Evaluation of Plasmalogen levels with electronic cigarette use. The box plots show the distribution of plasmalogen levels in female tobacco non users (Non User), electronic cigarette users (EC) and tobacco cigarette (TC) smokers. The horizontal line in each box represents the median, the lower and upper boundaries of the boxes the interquartile range, and the ends of the bars 1.5 times the interquartile range. *P < 0.05

Since changes in individual lipids can impact health and disease states without necessarily changing an entire lipid class, we compared lipid species across groups using ANOVA with group corrections. Male participants showed a significant change in a number of specific lipids including individual CE, PC, and HCER mostly driven by an increase in TCIG group when compared with ECIG and non-smokers (Fig. 6a). These results suggest that male ECIG users have a lipid profile that is largely indistinguishable from non-smokers. Similar to male counterparts, female participants showed significant changes in free fatty acids driven by TCIG smokers with no major changes between ECIG or non-smokers for FFA (Fig. 6b). Intriguingly however, a number of plasmalogen species PE (P-16:0/18:2) and PE (P-18:1/18:2) were significantly lower in female ECIG users compared with non-smokers and TCIG smokers (ANOVA followed by multiple comparisons of means at family-wise error rate 0.05) (Fig. 6b). Comparison of female ECIG and non-smokers showed significant mean differences in total plasmalogens (ANOVA, p = 0.02649); with non-smoker mean 73.1 nmol/ml (95% CI, 59.98 to 86.31) and ECIG mean 54.26 nmol/ml (95% CI, 44.61 to 63.90) (Fig. 7). This, results indicate that ECIG use is associated with alterations in lipid species known to be physiologically relevant and that changes associated with ECIG use are independent of and do not necessarily recapitulate changes associated with TCIG smoking.

## Discussion

Our study is one of the first to interrogate changes in lipid species associated with tobacco products in healthy humans. We observed increases in cholesterol esters and fatty acid species with TCIG smoking which is consistent with previous studies [19]. ECIG use, however, does not appear to be associated with dramatic changes in lipid composition although significant alterations in a number lipid species were observed and appear to be independent of nicotine use. Although males and females are known to have differences in lipid species composition, the clinical significance of the sex-specific differences observed in our study are unclear. Intriguingly, a murine study found changes in lipid composition associated with ECIG use specifically using female mice [9], however serious complications associated with ECIG use have been reported in predominantly male population although the overall numbers have been small. Although this is one of the largest ECIG studies to date, our sample size is relatively small and tests in larger cohorts of subjects are needed to confirm our findings. In addition, we measured lipid changes in plasma but it is conceivable that ECIG use is associated with local (alveoli) changes in lipids that are not transmitted in plasma or contributions from other organs may be at play. Although most of our study participants reported no medications and oral contraceptive use was reported as minimal (5 subjects) it is possible that other medications or supplements may be underreported and could interfere with lipidomic measurements.

Female ECIG users had significantly lower plasmalogen levels compared with non-smokers. Plasmalogens are a class of lipid species that are critical for peroxisome function [13]. Inherited plasmalogen deficiency is associated with Zellweger spectrum and Rhizomelic chondrodysplasia punctate, a syndrome characterized by bone shortening, neurodevelopmental abnormalities, and, importantly, recurrent respiratory tract infections [13]. In addition, a number of studies have reported associations between low plasmalogen levels and respiratory diseases [20]. Plasmalogens are thought to contribute to the proper function of surfactant. Additionally, the lungs are uniquely exposed to ROS and plasmalogens are known to be protective against oxidative stress. Whether the alternations we observed meaningfully contribute to ECIG associated lung injury or other comorbid conditions remains unknown. Future studies will explore this question in greater detail.

## Conclusion

Out work finds that ECIG use is not associated with global changes in lipid composition but surprisingly specific alterations in serum lipids known to influence normal lung function were observed and this pattern was independent of tobacco use.

## Data Availability

Not applicable.

## Abbreviations

ECIGS: Electronic cigarettes
TCIGS: Tobacco cigarettes
